# Subclinical cardiac dysfunction in pediatric kidney transplant recipients identified by speckle-tracking echocardiography

**DOI:** 10.1007/s00467-022-05422-7

**Published:** 2022-02-15

**Authors:** Adrienn Bárczi, Bálint Károly Lakatos, Mónika Szilágyi, Éva Kis, Orsolya Cseprekál, Alexandra Fábián, Attila Kovács, Attila J. Szabó, Béla Merkely, Paolo Salvi, György S. Reusz

**Affiliations:** 1 1st Department of Pediatrics, Semmelweis University, Bókay János Str. 53-54, Budapest, 1083 Hungary; 2 Heart and Vascular Center, Semmelweis University, Budapest, Hungary; 3 Gottsegen György Hungarian Institute of Cardiology, Budapest, Hungary; 4 Department of Surgery and Transplantation, Semmelweis University, Budapest, Hungary; 5 IRCCS, Cardiology Unit, Istituto Auxologico Italiano, Milan, Italy

**Keywords:** Cardiovascular disease, Hypertension, Longitudinal strain, Kidney transplant children, Speckle-tracking echocardiography

## Abstract

**Background:**

Kidney transplantation (KTx) improves prognosis in children with kidney failure; still, these patients are prone to cardiovascular damage due to multiple risk factors. Our aim was to assess myocardial structure and function in pediatric KTx by conventional and speckle-tracking echocardiography (STE) in association with established cardiovascular risk factors.

**Methods:**

Forty-two KTx and 39 healthy age- and gender-matched children were evaluated. KTx recipients were further categorized according to the control of hypertension assessed by 24-h ambulatory blood pressure monitoring (ABPM). Subjects underwent pulse wave velocity (PWV) measurement, conventional echocardiography, and 2-dimensional STE. Left and right ventricular (LV, RV) global longitudinal strain (GLS), and LV circumferential strain (GCS) were measured. Glomerular filtration rate (eGFR) was calculated according to the Schwartz formula.

**Results:**

KTx patients had increased blood pressure and arterial stiffness. LV ejection fraction (EF) was preserved along with elevated LV mass index (LVMi) while LVGLS was significantly lower, whereas LVGCS and RVGLS were increased in KTx. Uncontrolled hypertensives had lower LVGLS compared to those with controlled hypertension. Using multiple forward stepwise regression analysis, 24-h SBP and relative wall thickness (RWT) were independent determinants of LVMi, whereas antihypertensive therapy, eGFR, and HOMA-IR were independent determinants of LVGLS.

**Conclusions:**

Cardiac morphology and function show distinct changes after KTx. Along with comparable ventricular volumes, LV hypertrophy and subclinical myocardial dysfunction are present. Control of hypertension and kidney graft function are major factors of LV performance. STE may be useful to reveal early myocardial dysfunction in pediatric KTx.

**Graphical abstract:**

A higher resolution version of the Graphical abstract is available as Supplementary information.

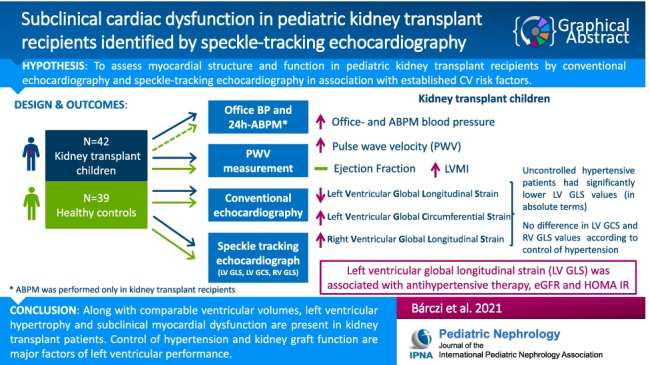

**Supplementary Information:**

The online version contains supplementary material available at 10.1007/s00467-022-05422-7.

## Introduction

Children with chronic kidney disease (CKD) are at high risk of developing cardiovascular diseases [1]. Kidney transplantation (KTx) offers improved patient survival and quality of life compared to chronic dialysis; however, pediatric KTx recipients still remain at risk of higher rates of cardiovascular (CV) events throughout their lives. In fact, sudden cardiac arrest, arrhythmias, or myocardial infarction are responsible for 9–45% of premature deaths in the pediatric kidney allograft population [2].

Beyond the pretransplant metabolic changes associated with uremia, children are also exposed to transplant-associated factors, such as immunosuppressive treatment with its cardiometabolic effects and a gradually decreasing graft function, as well as risk factors such as hypertension, increased arterial stiffness [3], post-transplant diabetes, or dyslipidemia.

Early detection of CV dysfunction in the pediatric population is of crucial importance in the prevention of later CV events. Subtle alterations in cardiac structure are already present in children with chronic kidney disease (CKD) with no or mild symptoms [4]. Left ventricular hypertrophy (LVH) assessed by conventional echocardiography is common in CKD as well as following KTx, and frequently associated with poor CV outcome in adults [5]. In children, while LVH is typically asymptomatic, it is still considered as a surrogate marker for target organ damage [6].

In addition to alterations in cardiac geometry, systolic and diastolic function of the heart can also be impaired even if conventional functional features such as ejection fraction (EF) are preserved [7, 8]. Two-dimensional (2D) speckle-tracking echocardiography (STE) is an advanced method for assessing myocardial deformation and mechanics. This technique is based on the tracking of natural acoustic reflections, the so-called speckles, on a grey-scale echocardiographic image [9]. It allows the assessment of regional and global myocardial deformation in different anatomically relevant axes, expressed as relative shortening and lengthening (“strain”). Global longitudinal strain (GLS) has been proven to be a more sensitive marker of myocardial dysfunction compared to EF and is considered as a robust predictor of adverse cardiac events in a wide variety of diseases [10]. Although GLS is an important marker of myocardial contractile function, data on its significance in pediatric KTx patients, however, is sparse.

Accordingly, the aim of the present study was to assess myocardial structure and function in pediatric KTx recipients by conventional echocardiography and STE in association with established CV risk factors.

## Methods

### Study population

Between January 2017 and June 2018, 42 kidney transplant recipients transplanted in childhood (age 14.0 ± 3.3 years, range 7.21–18.92 years) were enrolled in this single-center cross-sectional study. Inclusion criteria were a functioning allograft and no history of overt cardiovascular anomalies. Age- and gender-matched healthy children without any chronic diseases were enrolled as controls (*n* = 39, age 13.7 ± 3.5 years, range 8.11–18.55 years).

Demographic data, etiology of primary kidney disease, history of dialysis and transplantation, graft source, and immunosuppression treatment were reviewed from medical charts. Clinical and laboratory data were collected on the day of the echocardiographic measurements. Laboratory evaluation was performed only in KTx children. Serum levels of creatinine, lipid profile (cholesterol, HDL, LDL, triglycerides), fasting glucose, and serum insulin level were assessed by routine laboratory methods. eGFR was calculated according to the Schwartz formula [11]. The homeostatic model assessment for insulin resistance index (HOMA-IR) was computed with the formula: fasting plasma glucose (mmol/l) times fasting serum insulin (mU/l) divided by 22.5.

### Anthropometric data and blood pressure measurements

Height and weight and BMI were expressed as Z-scores according to Hungarian pediatric growth charts [12]. Body surface area (BSA) was estimated using the Mosteller formula [13].

Office brachial blood pressure (OBP) and heart rate were measured by a validated automated oscillometric device (Omron M4, Omron Healthcare, Kyoto, Japan) in sitting position using appropriate cuff sizes [6]. Heart rate and systolic and diastolic blood pressures were calculated as the mean of 3 measurements. Absolute blood pressure values are presented along with Z-scores for age and height [6].

In KTx patients, 24-h ambulatory blood pressure monitoring (ABPM) was performed by using a validated oscillometric device (ABPM-04, Meditech Kft., Budapest, Hungary). ABPM was set to record blood pressure in 20-min intervals during the day and every 30 min during the night. Mean 24-h as well as daytime and nighttime systolic blood pressure (SBP) and diastolic blood pressure (DBP), mean arterial pressure (MAP), and nocturnal systolic and diastolic blood pressure fall were recorded. ABPM data were expressed as Z-score for sex and height [14]. The diagnosis of hypertension was based on 24-h ABPM recordings and was defined as SBP and/or DBP equal to or exceeding the 95th percentile for gender, height, or the use of antihypertensive medication.

Hypertensive children were further classified according to the control of hypertension. Uncontrolled hypertension was defined as SBP and/or DBP values exceeding the 95^th^ percentile for height in patients with or without antihypertensive medication. In patients with controlled hypertension, both SBP and DBP were below the 95^th^ percentile for height and patients were taking antihypertensive medication. The normotensive group included patients with SBP or DBP below the 95^th^ percentile without taking antihypertensive medication. Nocturnal dipping was defined as nocturnal decrease in BP measured by ABPM. Patients with dipping below 10% were considered as non-dippers [15].

Based on OBP and ABPM results, patients having normotensive OBP values but hypertension on ABPM were classified as masked hypertensives, whereas patients with white coat hypertension had elevated OBP but normotension on ABPM [16].

Finally, based on ABPM measurements, patients with BP exceeding 95^th^ percentile at night, but with normal blood pressure during the day were categorized as having isolated nocturnal hypertension while patients with BP exceeding 95^th^ percentile during the day, but with normal blood pressure during the night were categorized as having isolated daytime hypertension. Patients with both daytime and nocturnal hypertension considered to have sustained hypertension [16, 17].

### Arterial stiffness

Aortic pulse wave velocity (PWV) was assessed by applanation tonometry (PulsePen device, DiaTecne, Milan, Italy) as described previously [18, 19]. Three consecutive measurements were recorded. Aortic PWV was calculated as the distance between the carotid and femoral sampling sites divided by the time difference between the rise delay of the distal and proximal pulse according to the R wave belonging to the ECG qRs complex. Age-, sex-, and height-specific Z-scores were calculated using our previously established normative data [18].

### Conventional and tissue Doppler echocardiography

Echocardiographic examinations were performed with a Vivid E95 ultrasound system equipped with a M5SC-D phased-array transducer (GE Vingmed Ultrasound, Horten, Norway). All measurements were performed according to current guidelines [20]. There were no patients with suboptimal image quality who should have been excluded from further analysis. Left ventricular (LV) wall thicknesses and diameters were evaluated in parasternal long-axis view at the level of the mitral valve coaptation using the 2D-guided M-mode technique. Relative wall thickness (RWT) was calculated as end-diastolic posterior wall thickness multiplied by 2 and divided by LV end-diastolic internal diameter. LV mass (LVM) was calculated using the Devereux formula [21]. LV end-diastolic (EDVi) and end-systolic volume indices (ESVi) indexed to BSA were measured from apical 4- and 2-chamber views by using the Simpson method to assess LVEF. LVH was defined as LV mass index (LVMi) equal to or exceeding the age-specific 95th percentile according to Díaz et al. [22]. LV geometry was considered to be normal when LVMI was < 95th percentile and RWT ≤ 0.42; concentric remodeling when LVMI was < 95th percentile and RWT ≥ 0.42; concentric hypertrophy when LVMI was ≥ 95th percentile and RWT ≥ 0.42; and eccentric hypertrophy when LVMI was ≥ 95th percentile and RWT < 0.42 [22].

Right ventricular (RV) basal diameter was measured as the transversal diameter at the basal third of the RV cavity on the apical 4-chamber view. Tricuspid annular plane systolic excursion (TAPSE) was measured using M-mode tracing of the tricuspid annulus at the free wall on the apical 4-chamber view.

Pulsed wave Doppler interrogation at the level of the mitral valve coaptation was obtained to determine early (E) and late diastolic (A) peak LV inflow velocities and their ratio (E/A). Pulsed wave Tissue Doppler Imaging (TDI) was used to measure systolic (s’), early (e’), and late diastolic (a’) velocities at the mitral lateral and medial annuli. The ratio of E-wave velocity to averaged e’ velocities of the mitral medial and lateral annuli was calculated as an estimate of LV filling pressures [23].

### 2D speckle-tracking echocardiography

STE is based on B-mode gray-scale tracking of 2D “speckles” or “kernels.” Using the end diastolic dimensions as a surrogate for the original length and reference point, these spots can be tracked as they move through the cardiac cycle to determine the degree of strain. Strain is a dimensionless parameter that represents the fractional change in the length of a myocardial segment in systole, where the longitudinal and circumferential shortening during myocardial contraction is represented by negative values and is expressed as a percentage (%). Since the myocardium contracts longitudinally and circumferentially during systole, these values are negative percentages, more negative values indicating better cardiac contractility, and accordingly if absolute values are considered, lower values of strain parameters implying dysfunction.

STE was performed with a dedicated software (Cardiac Perfomance Analysis, TomTec Imaging Systems GmbH, Unterschleißheim, Germany) using ECG-gated grey-scale cine-loops of three to five consecutive cardiac cycles by one experienced operator (A.B.). The endocardial surface of the given chamber was manually contoured on the end-diastolic frame. The algorithm automatically traces the endocardium throughout the cardiac cycle and calculates strain values. LVGLS was calculated using apical 2-, 3-, and 4-chamber views while global circumferential strain (GCS) was assessed from parasternal short axis views at the level of mitral valve, papillary muscles, and the apex. RVGLS was measured from the apical 4-chamber view [24]. Segments with inadequate tracking quality were excluded from further analysis.

Intra- and interobserver variability values of the key STE parameters showed appropriate agreement. The intra- and interobserver variability for LVGLS was 0.9481 (correlation coefficient of concordance, CCC), 2.7% (coefficient of variation, CV), and 0.9377 (CCC), 3.2% (CV) respectively, while for RVGLS, the same parameters were 0.8894 (CCC), 4.9% (CV), and 0.8246 (CCC), 5.2% (CV) respectively.

### Statistical analysis

Statistical analysis was performed using STATISTICA version 13.4 (TIBCO Software Inc, Palo Alto, CL). The Shapiro–Wilk test was used to detect the normality of data distribution. Variables are expressed as means ± SD, median (interquartile ranges), or percentage as appropriate. Categorical variables were analyzed using chi-square or Fisher’s exact test. Student’s *t*-test or Mann–Whitney *U*-test was used for comparing continuous variables for two groups according to normality. One-way ANOVA or Kruskal–Wallis test was used to compare multiple groups. Correlations between variables were evaluated by Pearson’s rank correlation test or non-parametric Spearman correlation. Stepwise multiple linear regression analyses were performed to assess the independent variables affecting LVMi and LVGLS. The *p* values reported are two-sided and taken to be significant at < 0.05. Inter- and interobserver variability was assessed by calculation of coefficient of variation (CV) and by Lin’s concordance correlation coefficient (CCC). Ten subjects (5 KTx group, 5 control) were re-analyzed in a blinded fashion by the operator of the initial measurements (A.B.), and the same subjects were also analyzed by a second operator (A.F.).

## Results

### Patient characteristics

General characteristics of the study groups are summarized in Table 1.

**Table 1:** General characteristics of the study groups

Parameters	KTx group (*n* = 42)	Control group (*n* = 39)	*p*
N (males/females)	27/15	22/17	0.50
Age (years)	14.0 ± 3.3	13.7 ± 3.5	0.71
Height (cm)	**151.4 ± 17.7**	**161.4 ± 18.9**	**0.01**
Height Z-score	**–1.15 ± 1.37**	**0.73 ± 1.31**	** < 0.001**
Weight (kg)	50.6 ± 17.6	50.7 ± 16.1	0.97
Weigh Z-score	0.03 ± 0.93	0.22 ± 0.70	0.30
BMI (kg/m^2^)	**20.0 (14.0–33.7)**	**18.0 (15.3–27.1)**	**0.04**
BMI Z-score	0.16 (–1.03–3.48)	–0.07(–1.14–1.59)	0.10
eGFR (ml/min/1.73 m^2^)	68.42 ± 23.83	–	
CKD stages (n/%)			
I	7 (16.6%)	–	
II	23 (54.7%)	–	
III	9 (21.4%)	–	
IV	3 (7.1%)	–	
HOMA-IR	2.34 (0.21–11.09)	–	
Ca × P (mmol^2^/l^2^)	3.22 (1.91–5.04)	–	
PTH (pg/ml)	74 (31–919)	–	

Data are presented as mean ± SD, median (interquartile range) or number of patients

Values with significant difference are presented in bold

*KTx* kidney transplantation, *N* number, *Z-score* standard deviation score, *BMI* body mass index

There were no significant differences with regard to age, gender, and weight Z-score between the KTx and control groups, while KTx patients were significantly shorter compared to controls.

Indications for KTx were congenital anomalies of the kidney and urinary tract (*n* = 13), focal segmental glomerulosclerosis (*n* = 10), polycystic kidney disease (*n* = 4), nephronophthisis (*n* = 4), VACTERL syndrome (*n* = 2), Denys–Drash syndrome (*n* = 2), ischemic kidney injury (*n* = 2), Alport syndrome (*n* = 1), Wegener’s granulomatosis (*n* = 1), tubulointersitial nephritis (*n* = 1), and unknown etiology (*n* = 2). The mean age at transplantation was 9.0 ± 3.7 years. Thirty-eight children received their first graft, while 4 had a second transplantation. Seventy-one percent of the patients received kidneys from cadaveric donors; the prevalence of living related kidney donation was 12/42 (29%); 32 patients required hemo- and/or peritoneal dialysis prior to transplantation (median time at dialysis was 11.1 months); 10 patients were transplanted preemptively. The mean GFR was 68.42 ml/min/1.73 m^2^; most patients were in CKD stage II, with only 3 in CKD stage IV.

Transplant recipients were on standard immunosuppression with tacrolimus and mycophenolate mofetil (*n* = 36); steroid was added in 10 patients. Two patients were treated with a combination of tacrolimus and steroid; 1 child was on tacrolimus monotherapy. mTOR inhibitor sirolimus was administered in 2 children in combination with mycophenolate mofetil.

### Blood pressure, arterial stiffness and echocardiographic parameters

Blood pressure, arterial stiffness, and characteristic echocardiographic parameters of the study groups are shown in Table 2.

**Table 2:** Blood pressure, arterial stiffness, and characteristic echocardiographic parameters of the study groups

Parameters	KTx group (*n* = 42)	Control (*n* = 39)	*p*
Office SBP (mmHg)	118 (99–149)	106(92–124)	** < 0.001**
Office SBP Z-score	0.94(–0.59–3.76)	0.08(–1.25–1.82)	** < 0.001**
Office DBP (mmHg)	66 (50–92)	61 (51–76)	**0.001**
Office DBP Z-score	0.44(–1.2–3.32)	–0.27(–1.71–1.37)	** < 0.001**
PWV (m/s)	5.28 ± 0.71	5.11 ± 0.77	0.55
PWV Z-score for height	0.60 ± 1.09	0.01 ± 0.92	**0.01**
RWT (%)	0.36 ± 0.05	0.31 ± 0.04	** < 0.0001**
LVMi (g/m^2^)	36.82 ± 6.80	27.91 ± 5.61	** < 0.0001**
LVEDVi	51.53 ± 13.55	52.85 ± 8.67	0.35
LVESVi	19.88 ± 6.84	20.17 ± 3.72	0.30
EF%	61.02 ± 5.85	61.75 ± 3.22	0.49
TAPSE (mm)	23.42 ± 3.55	24.18 ± 3.31	0.32
Transmitral E wave (cm/s)	103.02 ± 17.16	104.59 ± 12.41	0.64
Transmitral A wave (cm/s)	68.54 ± 16.56	61.18 ± 15.22	**0.04**
E/A ratio	1.57 ± 0.42	1.8 ± 0.46	**0.02**
DT (ms)	168.64 ± 30.11	162.59 ± 31.63	0.38
Mitral lateral anulus s’ (cm/s)	10.47 ± 2.71	11.24 ± 2.22	0.18
Mitral lateral anulus e’ (cm/s)	15.98 ± 3.05	19.54 ± 3.11	** < 0.0001**
Mitral lateral anulus a’ (cm/s)	6.49 ± 1.63	6.81 ± 1.82	0.43
Mitral medial anulus s’ (cm/s)	8.29 ± 1.36	9.11 ± 1.23	**0.008**
Mitral medial anulus e’ (cm/s)	11.31 ± 2.27	15.38 ± 2.53	** < 0.0001**
Mitral medial anulus a’ (cm/s)	7.43 ± 1.99	7.08 ± 1.48	0.39
E/e’ average ratio	7.74 ± 1.80	6.07 ± 1.04	** < 0.0001**
LV GLS	–20.64 ± 2.02	–21.78 ± 2.14	**0.002**
LV GCS	–26.54 ± 4.01	–24.86 ± 3.78	**0.03**
RV GLS	–24.34 ± 3.79	–22.39 ± 2.59	**0.01**

Data are expressed as mean ± SD

Values with significant difference are presented in bold

*KTx* kidney transplantation, *Z-score* standard deviation score, *SBP* systolic blood pressure *DBP* diastolic blood pressure, *PWV* pulse wave velocity, *LVESVi* left ventricular end-systolic volume index, *LVEDVi* left ventricular end-diastolic volume index, *RWT* relative wall thickness, *LVMi* left ventricular mass index, *EF* ejection fraction, *TAPSE* tricuspid annular plane systolic excursion, *DT* deceleration time, *LV GLS* left ventricular global longitudinal strain, *LV GCS* left ventricular global circumferential strain, *RV GLS* right ventricular global longitudinal strain

Twenty-five patients were treated for hypertension. Antihypertensive medication consisted of calcium-channel blockers (*n* = 14), beta-blockers (*n* = 18), ACE-inhibitors (*n* = 6), and alpha-adrenergic blocking agents (*n* = 2). A majority of the patients were on monotherapy (*n* = 14); 11 patients received double or triple combinations of antihypertensive medications. Seven patients had new-onset hypertension according to ABPM and were not yet on antihypertensive medication.

#### Blood pressure and arterial stiffness

Z-scores of blood pressure as well as of PWV of the KTx group significantly exceeded those of the control group.

#### Conventional, tissue Doppler and speckle-tracking imaging

RWT and mean LVMi of KTx patients were significantly higher compared to the healthy subjects (*p* < 0.001)*.* LVH was present in 13 patients (30%), (concetric hypertrophy *n* = 4; eccentric hypertrophy *n* = 9); however, conventional indicators of LV and RV systolic function (LVEF, TAPSE) did not differ from controls. Conventional Doppler-derived transmitral E wave was similar to the healthy controls while A wave was significantly increased, and as a result, E/A ratio was significantly lower in KTx patients.

According to tissue Doppler measurements, mitral medial and lateral annular e’ velocities and mitral medial annular s’ velocities were significantly lower in KTx patients. Moreover, E/e’ ratio was found to be significantly higher in KTx patients.

Speckle-tracking analysis showed significantly decreased absolute value of LVGLS (lower absolute values of strain parameters indicate dysfunction), while the absolute value of LVGCS and RVGLS was significantly higher in KTx patients.

### Blood pressure control and its effect on myocardial parameters

To analyze the effects of hypertension on myocardial structure and function, hypertensive KTx patients were compared with normotensive KTx patients.

General characteristics, blood pressure, and PWV data of both KTx groups are shown in Table 3.

**Table 3:** General characteristics, blood pressure parameters, and arterial stiffness of the transplant patients classified according to blood pressure categories

Parameters	Controlled HT (*n* = 11)	Uncontrolled HT (*n* = 21)	no HT (*n* = 10)	*p* cHT vs ucHT	p cHT vs no HT	*p* ucHT vs no HT
N (males/females)	6/5	15/6	6/4	0.44	0.99	0.68
Age (years)	13.67 ± 3.63	14.90 ± 2.80	12.52 ± 3.13	0.29	0.40	0.06
Time on dialysis (months)	11.03 ± 12.05	11.76 ± 12.66	10.92 ± 13.09	0.87	0.98	0.86
Time since KTx (months)	59.76 ± 45.35	62.91 ± 48.20	58.06 ± 39.10	0.85	0.93	0.78
Height (cm)	149 ± 20	155 ± 15	144 ± 17	0.37	0.48	0.10
Height Z-score	–0.77 ± 1.34	–1.34 ± 1.40	–1.15 ± 1.34	0.27	0.53	0.71
Weight. kg	55.06 ± 19.57	53.62 ± 17.40	39.2 ± 11.24	0.81	**0.03**	**0.03**
Weigh Z-score	0.52 ± 0.94	–0.03 ± 0.95	–0.37 ± 0.68	0.10	**0.02**	0.33
BMI (kg/m^2^)	23.43 ± 5.87	21.57 ± 5.12	18.46 ± 2.99	0.31	**0.02**	0.10
BMI Z-score	0.91 ± 1.21	0.40 ± 0.97	–0.01 ± 0.73	0.17	**0.04**	0.28
eGFR (ml/min/1.73 m^2^)	79.25 ± 18.51	62.04 ± 27.22	69.53 ± 19.18	0.15	0.72	0.79
Brachial SBP	118 ± 9	123 ± 12	110 ± 6	0.22	0.09	**0.003**
Brachial SBP Z-score	1.30 ± 0.92	1.55 ± 1.28	0.81 ± 1.05	0.56	0.33	0.10
Brachial DBP	65 ± 9	73 ± 10	62 ± 4	0.02	0.36	**0.002**
Brachial DBP Z-score	0.40 ± 1.17	1.07 ± 1.11	0.08 ± 0.58	0.08	0.47	**0.01**
Heart rate/min	81 ± 9	78 ± 10	78 ± 10	0.38	0.47	0.98
PWV m/s	5.23 ± 0.74	5.42 ± 0.74	4.95 ± 0.54	0.47	0.36	0.09
PWV Z-score for height	0.56 ± 1.25	0.65 ± 1.04	0.38 ± 1.01	0.89	0.70	0.53
24 h systolic BP (mmHg)	115 ± 7	127 ± 9	107 ± 6	** < 0.001**	**0.03**	** < 0.001**
24 h systolic BP Z-score	0.61 ± 0.69	2.09 ± 1.20	–0.27 ± 0.64	** < 0.001**	**0.04**	** < 0.001**
24 h diastolic BP (mmHg)	65 ± 5	76 ± 6	64 ± 6	** < 0.001**	0.61	** < 0.001**
24 h diastolic BP Z-score	–0.12 ± 0.95	1.59 ± 1.09	–0.32 ± 1.05	** < 0.001**	0.66	** < 0.001**
Nocturnal systolic BP fall %	11.01 ± 5.80	5.16 ± 6.10	13.40 ± 3.29	**0.007**	0.34	** < 0.001**
Nocturnal diastolic BP fall %	15.85 ± 7.14	9.80 ± 8.90	19.57 ± 5.54	**0.04**	0.29	**0.003**

Data are expressed as mean ± SD

Values with significant difference are presented in bold

*HT* hypertension, *cHT* controlled hypertension, *ucHT* uncontrolled hypertension, *N* number, *KTx* kidney transplantation, *BMI* body mass index, *Z-score* standard deviation score, *SBP* systolic blood pressure, *DBP* diastolic blood pressure, *PWV* pulse wave velocity

Based on 24-h ABPM results, 11 patients had controlled and 21 had uncontrolled hypertension, while 10 patients were normotensive. Normotensive KTx patients were thinner and had significantly lower BMI than patients with hypertension. Thus, Z-scores were used for statistical comparison of the blood pressure groups.

No differences were observed in terms of age, time on dialysis, or time since transplantation as well as for PWV values among the KTx groups.

Accordingly, due to the definition of hypertension, ABPM-measured blood pressure of hypertensive KTx children was significantly higher compared to the normotensive KTx children. The 24-h SBP and DBP Z-scores of the uncontrolled hypertensive patients were significantly higher, greater than 2 and 1.5 Z-scores respectively. Controlled hypertensives had lower blood pressure, although for the 24-h SBP value, it was still significantly elevated, almost 1 SD higher than that of normotensive children. These clear-cut differences were masked when considering office BP data.

Both systolic and diastolic nocturnal dipping were significantly impaired in the uncontrolled group compared to the controlled hypertensives. The prevalence of non-dipping phenomenon was 81% in uncontrolled and 45% in controlled hypertensive patients.

Echocardiographic parameters according to blood pressure control are shown in Table 4.

**Table 4:** Echocardiographic parameters according to blood pressure category

Parameters	Controlled HT (n = 11)	Uncontrolled HT (n = 21)	no HT (n = 10)	*p* cHT vs ucHT	*p* cHT vs no HT	*P* ucHT vs no HT
RWT (%)	0.38 ± 0.07	0.35 ± 0.05	0.34 ± 0.03	0.24	0.12	0.51
LVMi (g/m^2^)	38.49 ± 7.64	38.59 ± 6.58	31.26 ± 2.17	0.96	**0.01**	**0.003**
LVEDVi	48.92 ± 10.78	53.66 ± 14.84	50.11 ± 14.19	0.36	0.84	0.50
LVESVi	18.15 ± 4.12	21.41 ± 8.19	18.71 ± 6.12	0.21	0.85	0.31
EF%	62.22 ± 5.60	59.47 ± 6.39	62.81 ± 4.50	0.21	0.81	0.14
TAPSE (mm)	24.18 ± 3.51	23.80 ± 3.48	21.80 ± 3.58	0.77	0.12	0.14
Transmitral E wave (cm/s)	109.72 ± 16.82	98.85 ± 18.69	104.40 ± 12.40	0.09	0.47	0.39
Transmitral A wave (cm/s)	67.09 ± 12.93	69.04 ± 19.31	69.1 ± 15.22	0.75	0.78	0.99
E/A	1.67 ± 0.36	1.52 ± 0.48	1.56 ± 0.35	0.35	0.57	0.79
DT (ms)	157.54 ± 24.92	178.38 ± 33.11	160.4 ± 23.55	0.06	0.82	0.11
Mitral lateral anulus s’ (cm/s)	10.13 ± 2.01	10.86 ± 2.95	10 ± 2.86	0.53	0.91	0.42
Mitral lateral anulus e’ (cm/s)	15.66 ± 3.31	16.41 ± 3.32	15.4 ± 2.41	0.56	0.85	0.40
Mitral lateral anulus a’ (cm/s)	6.30 ± 0.82	6.56 ± 1.69	6.5 ± 2.06	0.71	0.80	0.91
Mitral medial anulus s’ (cm/s)	8.27 ± 1.72	8.38 ± 1.24	8.14 ± 1.29	0.82	0.84	0.66
Mitral medial anulus e’ (cm/s)	11.63 ± 2.72	11.38 ± 2.11	10.83 ± 2.59	0.78	0.44	0.54
Mitral medial anulus a’ (cm/s)	7.49 ± 2.43	7.21 ± 1.69	7.81 ± 2.19	0.76	0.72	0.45
E/e’ average	8.61 ± 2.60	7.10 ± 1.16	8.13 ± 1.55	**0.02**	0.54	0.12
LV GLS	–21.54 ± 1.35	–19.34 ± 2.16	–20.87 ± 1.88	**0.003**	0.43	**0.04**
LV GCS	–26.23 ± 3.23	–26.02 ± 4.20	–28.05 ± 4.38	0.89	0.32	0.19
RV GLS	–24.74 ± 4.74	–23.30 ± 3.99	–25.87 ± 1.99	0.40	0.55	0.11

Data are expressed as mean ± SD

Values with significant difference are presented in bold

*HT* hypertension, *cHT* controlled hypertension, *ucHT* uncontrolled hypertension, *LVESVi* left ventricular end-systolic volume index, *LVEDVi* left ventricular end-diastolic volume index, *RWT* relative wall thickness, *LVMi* left ventricular mass index, *EF* ejection fraction, *TAPSE* tricuspid annular plane systolic excursion, *DT* deceleration time, *LV GLS* left ventricular global longitudinal strain, *LV GCS* left ventricular global circumferential strain, *RV GLS* right ventricular global longitudinal strain

LVMi was significantly higher both in controlled and uncontrolled hypertensive patients compared to the normotensive group, while RWT was not different between the groups. LVH was present in 13 patients (4 in the controlled and 9 in the uncontrolled group). In the controlled hypertensive group, 2 patients had concentric and 2 eccentric hypertrophy, while in the uncontrolled hypertensive group 7 had eccentric and 2 concentric hypertrophy (*p* = NS). There were no differences in LVEF and TAPSE between hypertensive and normotensive KTx patients, while deceleration time (DT) was tendentially increased in the uncontrolled hypertensive group.

According to speckle-tracking analysis, patients with uncontrolled hypertension had (in absolute terms) the lowest LVGLS values whereas controlled hypertensives had the highest values (Table 4).

### Control of hypertension according to ABPM subcategories

Based on OBP and ABPM results, 10 patients had masked and 4 had white coat hypertension. According to ABPM measurements, the 21 uncontrolled hypertensive patients could be further divided into sustained hypertension (combined day and night, *n* = 8), isolated daytime hypertension (*n* = 3), and patients with isolated nocturnal hypertension (*n* = 10). Comparing LVGLS results of the normotensive KTx patients (*n* = 21, patients with normal ABPM values with or without medication, LVGLS: –21.22 ± 1.62) to the ABPM subcategories, patients with isolated daytime HT had worse values (– 16.50 ± 0.89, *p* = 0.0001) followed by those with sustained hypertension (– 19.14 ± 1.99, *p* = 0.001), and those with isolated nocturnal HT (– 20.57 ± 1.66, *p* = NS).

Concerning the LVGCS values, patients with isolated daytime hypertension had the worst values (– 21.05 ± 2.34, *p* = 0.01), while the results for the sustained and isolated nocturnal hypertension categories (LVGCS – 26.54 ± 1.80 and – 27.10 ± 5.15 respectively) did not differ from the normotensive KTx group (LVGCS – 27.14 ± 3.86). The differences in RVGLS did not reach the level of significance.

### Correlations between echocardiographic parameters and clinical data

Univariate correlation analysis of LVMi and LVGLS of the KTx group is presented in Table 5.

**Table 5:** Univariate and multiple regression analysis of the determinants of LVMI and LV GLS

Univariate analysis	LVMI		LV GLS	
Variables	r	*p*	r	*p*
Age	0.14	0.34	0.22	0.15
Time on dialysis	–0.21	0.16	0.06	0.68
Time since Tx	0.02	0.86	0.26	0.09
BMI Z-score	**0.39**	**0.01**	0.12	0.42
GFR	–0.25	0.11	**–0.46**	**0.001**
Office SBP	0.003	0.98	0.13	0.37
Office DBP	–0.0006	0.99	0.16	0.30
24 SBP Z-score	**0.31**	**0.008**	**0.42**	**0.005**
24 DBP Z-score	0.18	0.25	0.27	0.07
Daytime SBP Z-score	**0.36**	**0.01**	**0.43**	**0.004**
Daytime DBP Z-score	0.25	0.09	**0.43**	**0.004**
Nighttime SBP Z-score	**0.32**	**0.04**	0.25	0.12
Nighttime DBP Z-score	0.24	0.13	0.25	0.11
Dipper status	0.29	0.07	0.01	0.92
Anti-HT status	0.08	0.59	**–0.30**	**0.05**
PWV SDS	0.23	0.12	**0.31**	**0.04**
LVMI	–	–	**0.47**	**0.001**
RWT	**0.47**	**0.001**	0.20	0.19
EF	–0.17	0.26	**–0.37**	**0.02**
E/A	–0.17	0.25	**–0.35**	**0.02**
E/e	0.11	0.47	0.11	0.48
Ca × P	0.19	0.21	0.21	0.17
HOMA-IR	0.21	0.21	**0.37**	**0.02**
Cholesterol	0.28	0.07	0.12	0.41
LDL	0.24	0.12	0.01	0.93
HDL	–0.05	0.75	–0.24	0.13
Multiple regression analysis	**ß**	***p***	**ß**	***p***
RWT	**0.45**	**< 0.001**	–	–
SBP Z-score	**0.37**	**0.005**	–	–
GFR	–	–	**–0.33**	**0.02**
HOMA-IR	–	–	**0.31**	**0.04**
Antihypertensive treatment	–	–	**–0.30**	**0.04**
LVMI	–	–	0.27	0.055

Cumulative R for multiple regression analysis of the determinants of LVMI, 0.33

Cumulative R for multiple regression analysis of the determinants of LV GLS, 0.41

Values with significant difference are presented in bold

*LVMI* left ventricular mass index, *LV GLS* left ventricular global longitudinal strain, *Tx* transplantation, *BMI* body mass index, *Z-score* standard deviation score, *GFR* glomerular filtration rate, *SBP* systolic blood pressure, *DBP* diastolic blood pressure, *HT* hypertension, *PWV* pulse wave velocity, *SDS* standard deviation score, *RWT* relative wall thickness, *EF* ejection fraction, *Ca* × *P* calcium phosphate product, *HOMA* homeostatic model assessment index, *LDL* low-density lipoprotein, *HDL* high-density lipoprotein, *HOMA-IR* homeostatic model assessment for insulin resistance index

LVMi correlated with Z-scores for BMI and systolic 24-h blood pressure as well as with RWT. Given the similarity between daytime/night-time ABPM values and the 24-h values, only the latter were included in the table.

LVGLS correlated with GFR, systolic Z-scores for 24-h ABPM, PWV Z-score, LVMi, EF, E/A, and HOMA-IR.

Of note, there was no correlation between age, time on dialysis or time since transplantation, the Ca × P product, PTH, and LVMi or strain parameters. Moreover, OBP values were not associated with either LVMI or LVGLS.

Twenty-four-hour SBP Z-score and RWT proved to be independent determinants of LVMi by multiple forward stepwise regression analysis, whereas the independent determinants of LVGLS were eGFR, HOMA-IR index, and the presence of antihypertensive therapy.

## Discussion

KTx is the treatment of choice for children with kidney failure with highly beneficial overall effects. However, complex mechanisms — including hypertension, increased arterial stiffness, components of metabolic syndrome, consequences of immunosuppressive therapy, and chronic graft dysfunction — are responsible for an elevated CV disease burden [1–4].

Increased CV risk may be assessed by various non-invasive techniques [4, 9]. In the current study, we aimed at evaluating KTx patients with a battery of non-invasive methods to show subtle differences that conventional methods may not detect.

Our KTx patients had increased blood pressure compared to controls, both with office measurements and 24-h ABPM. Hypertension — common in KTx — is one of the most important independent risk factors of developing LVH [25, 26]. The underlying mechanism of posttransplant hypertension is not fully understood. Among others, the use of immunosuppressive medications such as calcineurin inhibitors, steroids, chronic allograft dysfunction, and sympathetic overreactivity is responsible for elevated blood pressure. It is worthy of note, however, that in accordance with previous studies, 24-h ABPM was found more appropriate for defining blood pressure and hypertensive status [26–29] since only ABPM values correlated with the structural and functional parameters of the myocardium (see below).

In our study, KTx patients had significantly increased arterial stiffness (PWV Z-score), a well-known independent cardiovascular risk factor [29, 30] related to hypertension. Herein, PWV was correlated with blood pressure and LVGLS, but not with LVMi, the conventional measure of LVH. Vascular stiffness increases the speed and magnitude of reflected pulse waves, amplifying late systolic pressure and, thus, systolic load on the left ventricle resulting in injury to the subendocardium. In accordance with the report of Krishnasamy et al., LVGLS was associated significantly with arterial stiffness in patients with CKD 3–5 [31].

Increased muscular mass (that is higher LVMi) may cause diastolic dysfunction preceding impairment of systolic function [32, 33]. The significantly lower E/A ratio and early diastolic mitral annular velocities in our patients pointed to impaired LV relaxation. E/e’ as a surrogate measure of LV filling pressure was also significantly higher in KTx patients along with higher mitral valve inflow A-wave velocity, as reported previously [34, 35]. In turn, LV structural abnormalities were not accompanied by abnormalities in conventional parameters of systolic function such as EF or TAPSE (conventional systolic function parameter referring to RV longitudinal shortening) which were similar in both KTx patients and controls. In contrast, unlike conventional echocardiography, myocardial deformation imaging can detect early myocardial mechanical changes prior to alterations in ejection fraction in children with CKD [36]. Indeed, STE revealed that LVGLS was significantly impaired in transplanted patients compared to healthy controls, suggesting subclinical systolic dysfunction.

Moreover, similarly to adult patients with predominantly diastolic dysfunction and preserved LVEF, (absolute values of) LVGCS in the KTx group were significantly higher, suggesting, that the increased circumferential deformation compensates for the worsening of LVGLS, which together result in the preservation of the EF. These results point to the pathophysiological continuum that results in heart failure with preserved ejection fraction (HFpEF) in this population [37].

LVGLS was also found to be a predictor of outcome in the adult general population, patients with CKD and in adult KTx as well. In the Copenhagen City Heart Study, LVGLS predicted long-term risk of cardiovascular morbidity and mortality in a low risk general population [38]. In a prospective study by STE including 70 consecutive renal patients, Ravera et al. concluded that impairment of LV systolic function persists after starting dialysis and even in spite of successful kidney transplantation [39]. Reduced GLS is associated with increased risk of CV mortality both in CKD and KTx patients [40, 41]. STE measurements may also be prognostically important in children, provided this is confirmed by longitudinal studies with larger pediatric populations.

In KTx patients, there was a significant correlation between LVMi and BMI Z-score, ABPM, and RWT. The effect of blood pressure as well as BMI on LVMi is well established [42, 43]. KTx patients were significantly shorter than their healthy counterparts, explaining their somewhat increased, albeit still normal BMI levels.

While both the 24-h and separately assessed night and daytime SBP means were closely related with LVMi, the correlation with the dipper phenomenon showed only a trend (*p* = 0.07).

In contrast to LVMi, LVGLS was significantly associated with a larger number of parameters including GFR, ABPM, antihypertensive status, PWV, LVMi, EF, E/A, and HOMA index.

Since these various metabolic and cardiovascular markers are known to affect CV “wellness” in adults, LVGLS may be an early and sensitive indicator of myocardial damage in these pediatric patients, serving as an integrative parameter.

In our study, using stepwise multiple linear regression analysis, increased blood pressure was the major independent determinant of LVMi; while GFR, HOMA-IR index, and antihypertensive therapy were the independent determinants of LVGLS. These results are in good accordance with the study of Sgambat et al. who found that LVGLS was associated with hypertension, obesity, and metabolic syndrome in the posttransplant period, while higher GFR values appeared to be protective on LVGLS [44]. Interestingly, antihypertensive drug intake was a stronger determinant of LVGLS compared to direct markers of blood pressure. This may indicate that the long-term cardioprotective effects of antihypertensive treatment may be more significant than a simple “snapshot” of the current blood pressure status. Antihypertensive medications are reported to improve mechanical systolic and diastolic function in hypertensive heart disease [45] as well as longitudinal strain but not LVM, the latter being associated with lower blood pressure [46].

Insulin resistance after kidney transplantation is a multifactorial process. Increased BMI as well as the contrainsular effects of immunosuppressive agents like steroids, calcineurin inhibitors may contribute to impaired glucose metabolism. According to recent data, there is a relationship between insulin resistance and subclinical myocardial dysfunction assessed by STE in different states of disease [47]. An association between LVGLS and HOMA-IR has also been reported previously in obese children [48].

These results highlight that traditional “adult” CV risk factors also affect myocardial structure and function in high-risk pediatric patients. Little is known regarding right ventricular function of KTx patients. Interestingly, in the current study, RVGLS was significantly higher in transplanted patients compared to controls, which may be attributable to an early adaptive response to adverse LV remodeling. Since CKD is associated with chronic hypervolemia, increased RVGLS may be perceived as a “remnant” of adaptation to increased RV preload and filling pressures, which can result in increased RV deformation in the early stages [49]. It is also underpinned by the fact that E/e’ showed a statistically significant correlation with RVGLS.

Finally, when examining the efficacy and effects of blood pressure control in the KTx group, we found the latter to be suboptimal in a high proportion of our patients. The prevalence of hypertension was 76%, with controlled hypertension observed in only 34% of the KTx population. As highlighted above, OBP levels did not reflect the significant impact of blood pressure on myocardial structure and function revealed by 24-h ABPM. As reported in several pediatric and adult studies, ABPM values are better associated with target organ damage or graft function than office blood pressure [25, 26].

While the EF did not differ among the KTx subgroups, STE revealed subtle myocardial dysfunction since patients with uncontrolled hypertension had the significantly lowest LVGLS values, whereas controlled hypertensives exhibited the best values.

Considering OBP and ABPM data, classification of hypertension may be more accurate and better supported by 24-h monitoring revealing white coat and masked hypertension. Further, the lack of nocturnal dipping as well as the categories of isolated nocturnal and isolated daytime hypertension can only be detected using ABPM [16, 17]. Comparing ABPM and OBP data of 198 patients of the CKiD cohort study, Mitsnefes et al. found that 38% of children had masked hypertension and 18% had confirmed hypertension (both elevated casual and ambulatory BP). LVH was more common in children with either confirmed (34%) or masked (20%) hypertension compared with children with normal casual and ambulatory BP (8%) [16]. In our cohort, isolated nighttime and sustained hypertension was found in 19% and 23% of patients, respectively, while the prevalence of isolated daytime hypertension was lower (7%), similarly as reported by Düzova et al. [17]. McGlothan et al. also described the predominance of nocturnal hypertension in pediatric kidney allograft recipients [50].

The main advantage of ABPM is not only to detect hypertension but to provide a better control of day- and nighttime blood pressure of hypertensive patients, which is crucial in the maintenance of cardiovascular health [26]. The high proportion of hypertensive patients and the high percentage of non-dippers among both uncontrolled and controlled hypertensives may explain the higher LVMi compared to the non-hypertensive KTx patients. The similar proportion of increased LVMi among the hypertensive subgroups indicates that other factors may have a stronger influence on LVH; as a result the antihypertensive treatment and the blood pressure level attained were not sufficient to decrease the LVMi. This is also true when considering the subcategories of sustained, isolated daytime, and isolated nocturnal hypertension, which had similar values to the uncontrolled group (due to the overlap between categories). The group of masked hypertension was comparable to the uncontrolled group, while patients with white coat hypertension were rather similar to those with controlled hypertension.

### Limitations

This study provides insight into myocardial structural and functional changes in patients after KTx. However, certain important limitations should be highlighted. First, this is a single-center study with a relatively small number of cases. Due to the limited sample number overall, and especially after classifying patients into the individual blood pressure groups, the effect of etiology, immunosuppressive, or antihypertensive treatment (in particular the use of ACE inhibitors and ARBs) on cardiac structure, the category of myocardial hypertrophy and mechanics, could not be assessed in detail. Concerning calcium and phosphate metabolism and its influence on cardiac function, the Ca × P product and PTH were not associated with the results of STE; however, FGF23 — an important factor of myocardial dysfunction in CKD — was not examined. Moreover, the cross-sectional design of the study precludes establishing a causal relationship between the studied parameters.

In conclusion, pediatric KTx patients show several high-risk features in terms of CV disease, and these metabolic and hemodynamic factors are associated with changes in cardiac morphology and function. While conventional measures of LV and RV performance do not effectively reflect these changes, STE-derived strain parameters may conversely serve as sensitive markers of myocardial damage. Early detection of subtle alterations and targeted treatment may prevent later CV complications. All these data suggest the use of STE in children may be promising and its predictive value should be evaluated in longitudinal studies.

## Supplementary Information

Graphical abstract (PPTX 63.7 kb)
