# Supplementary material for: Subclinical cardiac dysfunction in pediatric kidney transplant recipients identified by speckle-tracking echocardiography
Source: Pediatr Nephrol. 2022 Feb 15;37(10):2489–501. doi: 10.1007/s00467-022-05422-7 (PMC9395460; doi:10.1007/s00467-022-05422-7)
Supplement: Supplementary file 1 — (PPTX 63.7 kb) [file 467_2022_5422_MOESM1_ESM.pptx]

## Slide 1
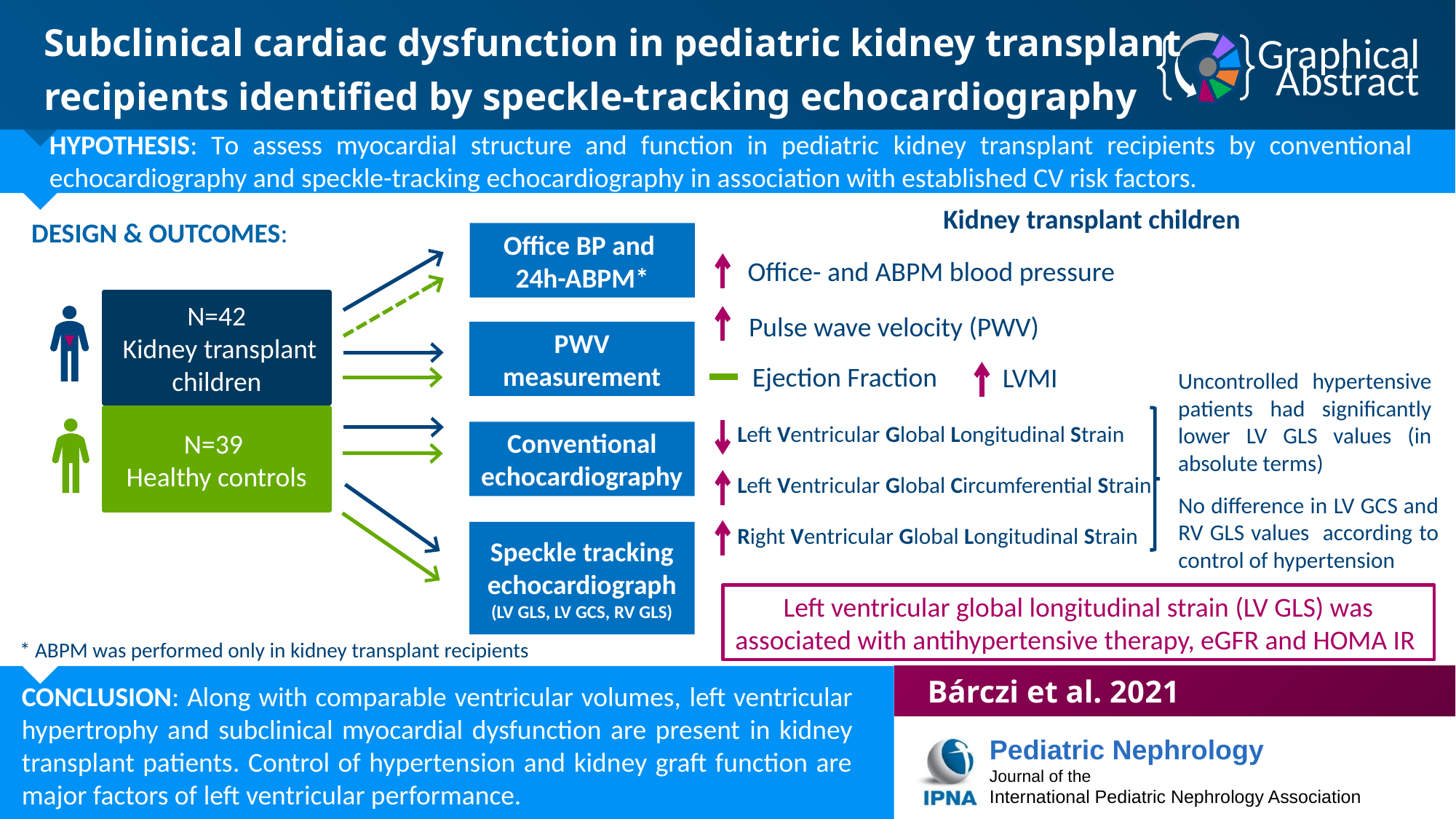

Subclinical cardiac dysfunction in pediatric kidney transplant recipients identified by speckle-tracking echocardiography
HYPOTHESIS: To assess myocardial structure and function in pediatric kidney transplant recipients by conventional echocardiography and speckle-tracking echocardiography in association with established CV risk factors.
Kidney transplant children
DESIGN & OUTCOMES:
Office BP and
24h-ABPM*
Office- and ABPM blood pressure
Pulse wave velocity (PWV)
LVMI
Left Ventricular Global Longitudinal Strain
Left Ventricular Global Circumferential Strain
Right Ventricular Global Longitudinal Strain
N=42
 Kidney transplant
children
PWV measurement
Ejection Fraction
Uncontrolled hypertensive patients had significantly lower LV GLS values (in absolute terms)
N=39
Healthy controls
Conventional echocardiography
No difference in LV GCS and RV GLS values according to control of hypertension
Speckle tracking echocardiograph
(LV GLS, LV GCS, RV GLS)
Left ventricular global longitudinal strain (LV GLS) was associated with antihypertensive therapy, eGFR and HOMA IR
* ABPM was performed only in kidney transplant recipients
Bárczi et al. 2021
CONCLUSION: Along with comparable ventricular volumes, left ventricular hypertrophy and subclinical myocardial dysfunction are present in kidney transplant patients. Control of hypertension and kidney graft function are major factors of left ventricular performance.
